# The completeness of the left atrial appendage amputation during routine cardiac surgery

**DOI:** 10.1186/s12872-023-03330-8

**Published:** 2023-06-20

**Authors:** Dejan Radakovic, Kiril Penov, Marc Lazarus, Nodir Madrahimov, Khaled Hamouda, Christoph Schimmer, Rainer G. Leyh, Constanze Bening

**Affiliations:** 1 Department of Thoracic and Cardiovascular Surgery, University Hospital Würzburg, Oberdürrbacher Strasse 6, 97080 Würzburg, Germany; 2 Department of Anaesthesiology, Intensive Care, Emergency and Pain Medicine, University Hospital Würzburg, Würzburg, Germany

**Keywords:** Left atrial appendage occlusion, Cut and sew technique, Atrial fibrillation

## Abstract

**Background:**

Left atrial appendage (LAA) is the origin of most heart thrombi which can lead to stroke or other cerebrovascular event in patients with non-valvular atrial fibrillation (AF). This study aimed to prove safety and low complication rate of surgical LAA amputation using cut and sew technique with control of its effectiveness.

**Methods:**

303 patients who have undergone selective LAA amputation were enrolled in the study in a period from 10/17 to 08/20. The LAA amputation was performed concomitant to routine cardiac surgery on cardiopulmonary bypass with cardiac arrest with or without previous history of AF. The operative and clinical data were evaluated. Extent of LAA amputation was examined intraoperatively by transoesophageal echocardiography (TEE). Six months in follow up, the patients were controlled regarding clinical status and episodes of strokes.

**Results:**

Average age of study population was 69.9 ± 19.2 and 81.9% of patients were male. In only three patients was residual stump after LAA amputation larger than 1 cm with average stump size 0.28 ± 0.34 cm. 3 patients (1%) developed postoperative bleeding. Postoperatively 77 (25.4%) patients developed postoperative AF (POAF), of which 29 (9.6%) still had AF at discharge. On 6 months follow up only 5 patients had NYHA class III and 1 NYHA class IV. Seven patients reported with leg oedema and no patient experienced any cerebrovascular event in early postoperative follow up.

**Conclusion:**

LAA amputation can be performed safely and completely leaving minimal to no LAA residual stump.

## Introduction

The left atrial appendage (LAA) is a protuberance of the left atrium, which represents a 2–4 cm large, tubular structure that has a narrow connection to the left atrium and rests against the free wall of the left ventricle [1]. The use of transoesophageal echocardiography has made clear imaging of the LAA possible, so that its size, shape, flow patterns, and content can be assessed in health and disease. The LAA normally has a pulsatile blood flow in the sinus rhythm, whereby stasis and thus clot formation are prevented [2]. If necessary, clot formation can be detected using transoesophageal echocardiography (TEE) [1].

Atrial fibrillation (AF) is the most common abnormal heart rhythm (arrhythmia) caused by disorganized atrial depolarizations without effective atrial contraction and irregular AV conduction [3]. Its occurrence after cardiac surgery is common with prevalence of 10–65% associated with higher risk of mortality [4]. Although paroxysmal AF after cardiac surgery is often self-limiting [5], many patients after cardiac surgery with recurrent AF without suitable anti-coagulation remain at risk for stroke [6]. Left atrial appendage can be ligated or amputated as part of ablation procedure for atrial amputation or oversewn from inside as concomitant procedure during mitral valve surgery [7]. In the recent years the interest in LAA increased and led to larger number of different LAA occlusion devices, endocardial and epicardial which as such lead to further increase of operational costs [8].

Despite the fact that 90% of thrombi in non-valvular AF and 60% in valvular AF develop in the LAA, and being on site during open heart surgery it still has not become routine part of surgical prophylaxis during most of cardiac surgery procedures [9]. Most surgeons avoid LAA amputation, if atrial ablation is not planed due to risk of bleeding, risk of injury to circumflex artery and concerns for hemodynamic consequences as natriuretic hormones are primarily produced in LAA [10].

In this study we explored the safety and invasiveness of LAA amputation and its completeness as concomitant procedure during routine open-heart surgery.

## Patients and methods

### Study design and patients

We identified total of 303 patients between 10/17 and 12/20 who had LAA amputation performed. After institutional ethics review committee approval all the patients presenting for routine cardiac surgery were screened for eligibility and assigned for concomitant LAA amputation. The reason is that the resection of the LAA is part of SEIDC trial performed in our institution with prospective patient inclusion and all the surgeons followed this protocol. We analysed the patients enrolled in trial regarding the clinical aspects and consequences of LAA amputation [11–13]. The study was approved by the University Hospital ethics committee (IRB approval: 143/17-sc 6.10.2017). The inclusion and exclusion criteria for the study are as follows: patients undergoing routine open-heart surgery for the first time were included, while patients with a history of previous cardiac surgery or severe adhesions were excluded. Both, patients with history of AF and those signed for non-atrial fibrillation cardiac surgery were included. All patients gave informed consent.

### Operative technique

After placing the patient under general anaesthesia and performing median sternotomy patient was placed on cardiopulmonary bypass. Concomitant to planed cardiac surgery procedure LAA amputation was performed with cardioplegic arrest at a convenient time during the operation at the operating surgeon’s discretion. Amputation is performed under direct visualization provided by heart luxation and its effectiveness is controlled and confirmed using intraoperative transoesophageal echocardiography. LAA is excided direct at the basis and oversewn using a double-layered technique with 4 − 0 polypropylene suture. Meticulous inspection for remnants of bleeding is required and pledgeted-reinforced extra 4 − 0 Prolene stitch was placed if necessary. Fibrin sealant Tisseel (Baxter Healthcare Corp., Deerfield, IL) was used at surgeon´s discretion to cover the suture line.

### Postoperative care and outcome variables

All patients were admitted to surgical intensive care unit and monitored with continuous telemetry. ECG telemetry was kept during the whole hospital stay until to discharge. 12-lead ECG was obtained directly after operation and at discharge. Postoperative atrial fibrillation (POAF) was defined as AF occurring after operation during the hospitalization time with no previous history of AF. If occurred, AF was treated with rate control, antiarrhythmic drug therapy (almost exclusively amiodaron therapy), or electric cardioversion if the medical therapy alone failed. Anticoagulation was initiated in patients with POAF persisting for > 48 h.

All patients received a routine preoperative echocardiographic examination (Philips Epiq 7, Philips Health System, Hamburg, Germany). Measurements and analysis were performed by trained echocardiography technician and board-certified cardiothoracic attending surgeon blinded to patients and in accordance with the standardized echocardiography protocol and the American Society of Echocardiography.

Transthoracic echocardiography was performed at admission and before discharge. Transoesophageal echocardiography was performed as standard procedure during each surgery. Measurements and analysis were performed by trained and by German Society for Anaesthesiology and Intensive Care Medicine certified attending anaesthesiologist and in accordance with the standardized echocardiography protocol. The primary outcome variable was completeness of amputation (residual stump < 1 cm) which was controlled directly after discontinuation of cardiopulmonary bypass using transoesophageal echocardiography and bleeding complications. Further on, we investigated incidence of postoperative cerebrovascular events (CVEs); this included transient ischemic attack. Secondary variables were incidence of myocardial infarction, deterioration of renal function, changes in echocardiographic measurements.

At 6 months follow up the patients were interviewed regarding CVEs, NYHA status and signs of volume overload (legs oedema).

### Statistical analysis

Continuous variables are presented as mean and standard deviation and categorical variables as numbers with percentage. The normality of the continuous variables was assessed with the Shapiro-Wilk test. The preoperative and postoperative data were compared using t-test for parametric and Wilcoxon’s signed-rank sum test for non-parametric data; and categorical variables are compared using the chi-square or the Fisher’s exact test. A P value of < 0.05 was considered significant. All statistics were performed using SPSS software (IBM SPSS Statistics for Windows, Version 23.0, IBM Corp., Armonk, NY, USA).

## Results

A total of 303 patients had LAA amputation performed concomitant to routine cardiac surgery procedure. Table 1. describes the preoperative and demographic data. Most of the patients included in the study were males 249 (81.9%), and average patient was 69.9 ± 19.2 years old. As described surgical operative time was 114.4 ± 38.9 min. on cardio-pulmonary bypass and 82.2 ± 27.7 min cross clamp time as all procedures were performed on cardioplegic arrested hearts. The procedure most often performed was aortocoronary bypass 286 (94.4%). Surgical ablation or maze procedure was performed in 40 (13.2%) patients, although preoperative AF was present in 72 patients (23.7%) and only 1 (0.3%) patient had left atrial appendage thrombus. On admission 29 (9.5%) patient had history of stroke and 14 (4.6%) history of TIA, whereas carotid artery stenosis was present in 47 (15.5%) patients. 120 (39.5%) of patients reported NYHA class III or IV at admission and euroscore II was 3.09 ± 12.11. The mean CHA2DS2-VASc score was 3.43 ± 1.63, with 213 males having a score higher than 1 and 50 females having a score higher than 2. The mean HAS-BLED score was 3.29 ± 0.99, with 65 patients having a score between 0 and 2 and 238 patients having a score higher than 2 as shown in Table 1.

**Table 1:** Demographic and clinical characteristics of the study population

***Demographics***	
Age	69.9 ± 19.2
Male – n (%)	249 (81.9)
BMI (kg/m^2^)	29.1 ± 4.3
***Cardiovascular risk factors***	
Smoking – n (%)	123 (40.5)
Arterial hypertension – n (%)	261 (85.9)
Hypercholesterinemia – n (%)	206 (67.8)
Diabetes – n (%)	113 (37.2)
Myokardial infarction – n (%)	48 (15.8)
Atrial fibrillation – n (%)	72 (23.7)
TIA – n (%)	14 (4.6)
Apoplex – n (%)	29 (9.5)
Carotid artery stenosis – n (%)	47 (15.5)
Chronic Kidney Disease - n (%)	191 (62.8)
Peripheral artery disease – n (%)	36 (11.8)
Obesity – n (%)	
overweight	128 (42.1)
class I obesity	89 (29.3)
class II obesity	21 (6.9)
class III obesity	5 (1.6)
*Presentation*	
Coronary heart disease (CAD) – n (%)	
1-CAD	11 (3.6)
2-CAD	62 (20.4)
3-CAD	216 (71.1)
NYHA class III-IV – n (%)	120 (39.5)
Euroscore II	3.09 ± 12.11
CHA_2_DS_2_-VASc Score	
≥ 2 (male) – n (% of male)	213 (85.5)
≥ 3 (female) – n (% of female)	50 (92.6)
HAS BLED Score	
0–2 – n (%)	65 (21.5)
> 2 – n(%)	238 (78.5)
***Procedure performed***	
Aortic-coronary bypass – n (%)	286 (94.4)
Aortic valve surgery – n (%)	29 (9.6)
Mitral valve surgery – n (%)	15 (5)
Tricuspid valve surgery – n (%)	10 (3.3)
Ablation procedures (MAZE)	40 (13.2)
Cross clamp time (min)	82.2 ± 27.7
CPB time (min)	114.4 ± 38.9

BMI – body mass index; TIA - transient ischemic attack; NYHA - New York Heart Association; CPB – cardio-pulmonary bypass

In our study, there were three patients requiring reoperation and only one patient who required acute reoperation due to bleeding from LAA stump. No deaths or strokes were reported during the postoperative course. Regarding postoperative data as shown in Table 2. we detected POAF in 77 (25.4%) patients of which total of 29 (9.6%) were discharged with AF. Postoperative bleeding occurred in 3 (1%) patients and only in one case (0.33%) it was connected to LAA amputation. In this 81 year old patient repeated cross clamping was needed and tear was repaired with two pericardial strips. There were no patients with postoperative ECG signs of myocardial ischemia and average postoperative CK-MB was 59.5 ± 37.9 U/l.

**Table 2:** Postoperative data

*Variable*	N = 303
Afib at discharge – n (%)	29 (9.6)
POAF – n (%)	77 (25.4)
Postoperative bleeding – n (%)	3 (1)
RBC transfussions – n (%)	1.3 ± 2.5
CK (U/l)	687.7 ± 441.2
CK-MB (U/l)	59.5 ± 37.9
LAA residual stump > 1 cm – n (%)	3 (1.0)
LAA residual stump size (cm)	0.28 ± 0.34
DOACs at discharge – n (%)	15 (4.9)
OAC at discharge - n (%)	51 (16.8)
Amiodaron at discharge - n (%)	37 (12.2)
NYHA class III-IV at 6 months follow up – n (%)	6 (1.9)
Leg oedema at 6 months follow up – n (%)	7 (2.3)
Cerebrovascular event at 6 months follow up – n (%)	0 (0)

POAF – postoperative atrial fibrillation; RBC – red blood cell; CK – creatin kinase: LAA – left atrial appendage; DOAC - directly acting oral anticoagulants; OAC – oral anticoagulants (warfarin)

**Fig. 1:**
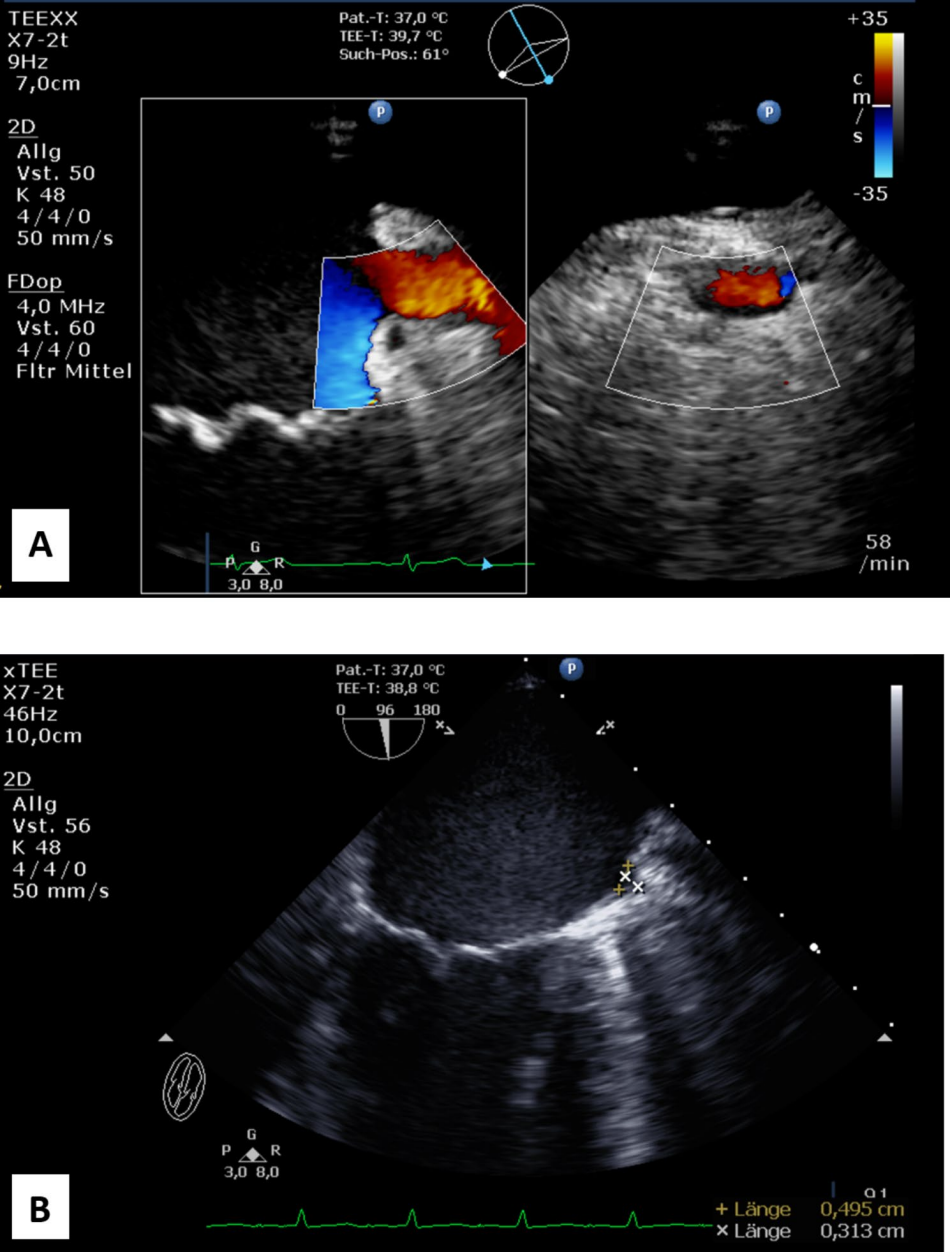
**A** – Before LAA amputation there is color low jet observed between the atrium and appendage suggesting persistent flow into left atrial appendage. **B** – after left atrial appendage is excised in same patient stump remains < 1 cm; measured 0.313 cm exactly

After LAA amputation was performed and cardiopulmonary bypass disconnected a final check of residual stump was performed and measured as shown in Fig. 1. In our patient group we identified 3 (1%) patients with LAA residual stump larger than 1 cm, with average stump size 0.28 ± 0.34 cm.

At 6 months follow up only 6 (1.9%) patients reported NYHA class III or IV, of which only one patient still in NYHA class IV. Seven patients (2.3%) had leg oedema and not one patient experienced cerebrovascular event during the first 6 months after surgery. In our study, 15 patients (4.9%) were on NOAC and 51 (16.8%) were on OAC according to standard practice at our institution. Specifically, patients were started on anticoagulation with heparin during the procedure, followed by oral anticoagulation with warfarin or DOACs postoperatively.

**Fig. 2:**
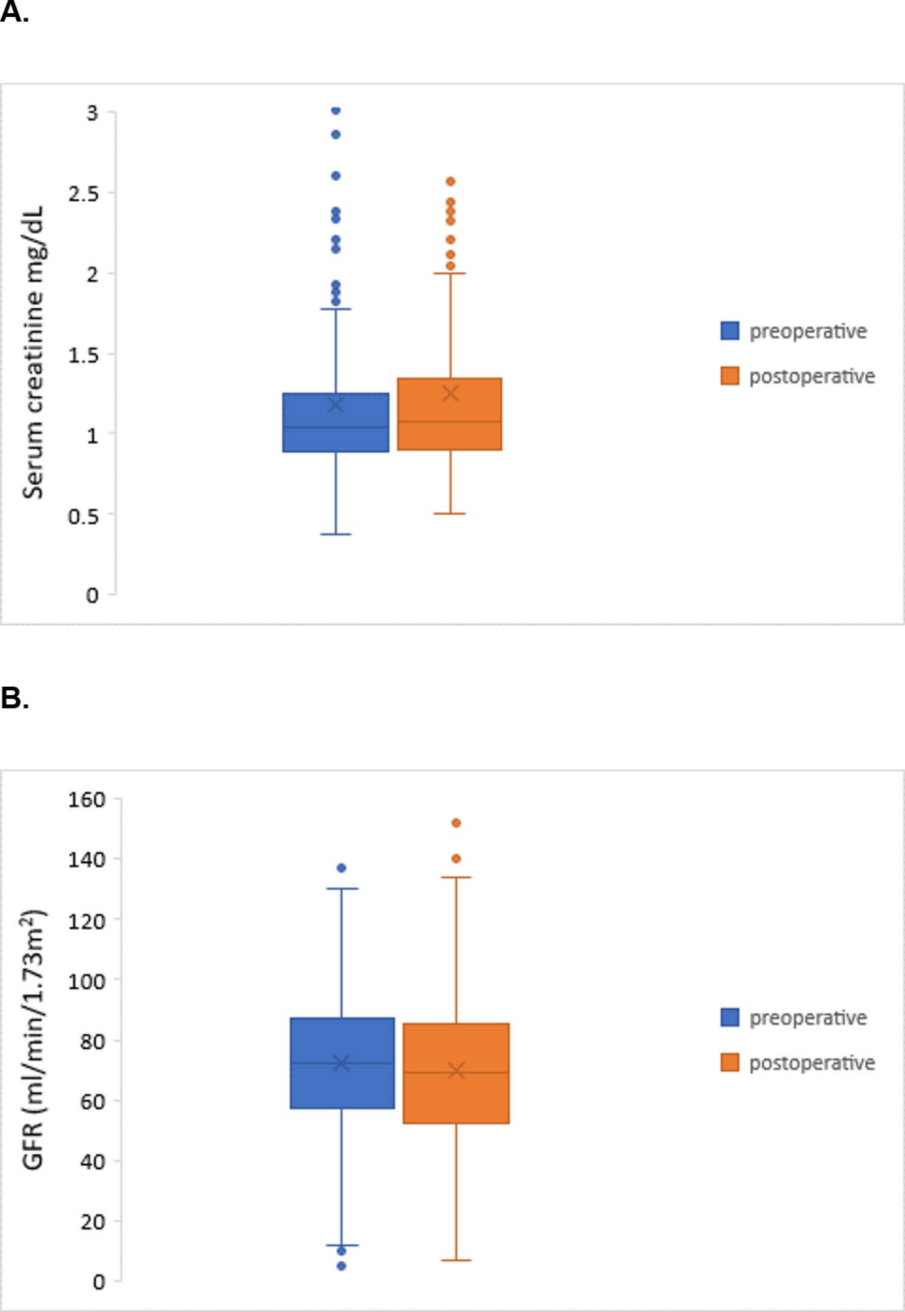
Kidney function before and after surgery. **A** – serum creatine levels. **B** - Glomerular filtration rate

In Fig. 2. we showed changes in renal function before and after surgery. We noticed slight reduction in GFR (72.28 ± 23.22 vs. 69.63 ± 24.52 mL/min/1.73m^2^, p = 0.02) and increase in serum creatine levels (1.18 ± 0.73 vs. 1.25 ± 0.67 mg/dl, p = 0.113).

Further on we analysed perioperative changes in transthoracic echocardiography which are listed in Table 3. Stroke volume reduced after surgery (92.4 ± 23.5 vs. 78.5 ± 19.5 ml, p = 0.000), while EF remained almost constant (49.9 ± 17.1 vs. 48.9 ± 11.3%, p = 0.113). TAPSE was significantly reduced after performed surgery (22.8 ± 4.5 vs. 14.4 ± 2.8 mm, p = 0.000). Most of the patients had mild degree of diastolic dysfunction already preoperatively with E/A ratio 0.98 ± 0.69 vs. 1.38 ± 0.73 (p = 0.000) and E/e´ 12.9 ± 7.9 vs. 13.1 ± 5.1 (p = 0.101). sPAP values remained in normal or slightly increased range (30.9 ± 11.4 vs. 28.7 ± 8.2 mmHg; p = 0.288). We noticed that NT-proBNP increased postoperatively (1.64 ± 4.3 vs. 2.38 ± 3.89 ng/ml, p = 0.000).

**Table 3:** Perioperative echocardiography data and NT-proBNP levels

	Preoperative	Postoperative	p value
EF (%)	49.9 ± 17.1	48.9 ± 11.3	0.113
Stroke volume (ml)	92.4 ± 23.5	78.5 ± 19.5	0.000
TAPSE (mm)	22.8 ± 4.5	14.4 ± 2.8	0.000
Left atrial volume (ml/m^2^)	59.2 ± 27.8	59.8 ± 41.4	0.863
Left atrial diameter (cm)	3.9 ± 2.1	3.5 ± 0.5	0.055
E/A ratio	0.98 ± 0.69	1.38 ± 0.73	0.000
E/e´ ratio	12.9 ± 7.9	13.1 ± 5.1	0.101
sPAP (mmHg)	30.9 ± 11.4	28.7 ± 8.2	0.288
LVIDd (cm)	4.74 ± 0.75	4.68 ± 0.51	0.249
NT-proBNP (ng/ml)	1.64 ± 4.3	2.38 ± 3.89	0.000

EF – ejection fraction; TAPSE - tricuspid annular plane systolic excursion; sPAP – systolic pulmonary artery pressure; LVIDd - left ventricular internal dimension-diastole; NT-proBNP - N-terminal pro-B-type natriuretic peptide

## Comment

In our study we showed in consequent transoesophageal echocardiography that complete resection of LAA with residual stump < 1 cm can be achieved in 99% of patients with stump size 0.28 ± 0.34 cm. Despite being more aggressive in LAA amputation we had no cases of postoperative myocardial infarction and bleeding risk with only 3 patients remained low. The surgical method used in our study was a cut and sew technique for LAA amputation. We chose this technique because it is a safe and effective method for LAA amputation and has been used in other clinical settings. However, it may have some disadvantages, such as the potential for incomplete closure of the LAA, which has not been thoroughly investigated so far. Overall, the cut and sew technique offers a viable option for LAA amputation during open-heart surgery. It is a cost-effective and safe procedure that showed a high rate of completeness of amputation in our study. As such cut and sew technique can be considered safe and sufficient, and more cost effective compared to staplers or occlusion devices.

Prevalence of AF after cardiac surgery ranges between 10 and 65% [14]. Most of these patients are asymptomatic, still if not receiving anti-coagulation after surgery they remain at risk of stroke. Since the patients being operated are getting older every day this represents a major problem in long term outcomes and increases risk of cerebro-vascular events [6]. Some of the problems regarding the oral anticoagulation are high risk of bleeding or insufficient anticoagulation during outpatient management [15, 16]. The new LAAOS III trial showed that concomitant LAA amputation reduced stroke in addition to oral anticoagulation. In this trial different techniques were used for left atrial appendage occlusion beyond cut and sew technique including stapler, closure devices and other approved techniques [17]. Therefore, LAA closure using surgical or percutaneous techniques is gaining tremendous interest. Knowing that most of cardiac emboli origin in left atrial appendage, most logical option for reduction of thromboembolic risk would be its exclusion from circulation during open heart surgery [8, 18]. Some of concerns are risk of bleeding using cut and sew technique, not complete exclusion and recanalization when oversewn from inside or occlude device migration during follow up [19, 20]. In most modern health systems a cost effectiveness of different techniques plays also an important role. Further on, leaving a residual stump larger than 1 cm after LAA excision remains of concern as it poses a risk of harbouring the thrombus [21]. In our study we found that only one patient (0.33%) had a postoperative bleeding connected to LAA amputation and only 3 patients (1%) had a residual stump larger than 1 cm. Moreover, electrical isolation of the LAA during surgical ablation cannot safely be achieved by suture and ligature techniques.

There are still some controversies regarding hemodynamic consequences after LAA amputation most important of it regarding secretion of natriuretic hormones which are primarily produced in LAA [10]. Initial animal experiments raised concerned due to impairment of mechanism to compensate the fluid overload, due to loss of atrium natriuretic peptides but showed no implication in humans [22]. Majunke et al. showed that LAA closure resulted in a significant increase in the ANP and BNP levels after the procedure [23]. This trend in the BNP is the same as in our study. It remains unclear if this is due to LAA amputation alone or if it might be influenced through other concomitant procedures performed. Nevertheless, on long term follow up BNP levels tend to get back to baseline levels up to 2 years postoperatively after LAA amputation having no or less clinically relevant effect on natriuretic peptide levels and severity of heart failure [24].

We also have not seen changes in left atrial volume in our patient series and left atrial diameter decreased after procedure. Of importance is also that NYHA class reduced at 6 months follow up and only 7 (2.3%) patients reported with leg oedemas. No significant change in EF was noted, but we did see higher E/A ratio which might be result of diastolic dysfunction of postcardioplegic myocardium. Also, we noted decreased TAPSE in postoperative control, this is well known phenomenon after cardiac surgery and opening of pericardial sack and postcardioplegic myocardium, so it is unclear if it is induced by LAA amputation alone [25].

It remains unclear if LAA exclusion can reduce or increase postoperative AF. Some studies advocate that LAA exclusion leads to less arrhythmogenic substrate and reduction of AF during follow up [26]. As such there are studies addressing the problem of prophylactic LAA exclusion during routine cardiac surgery, but most of them use heterogenic techniques such as closure from inside, using a stapler or device occlusion [27]. Direct suture is usually performed during mitral valve surgery either as purse string suture or as over-and-over suture. Direct suture has high risk of recanalization or incomplete closure which goes up to 30% [28]. Second method is ligation of LAA, but this can cause bleeding complications or central leakage [29]. Furthermore, in the last years clipping devices became available such as AtriClip device (AtriCure, Mason, OH, USA). The first reports show that AtriClip occlusion appears to be a reproducible and safe surgical method with a high success rate [30]. It is not always easy to apply device in patients with large LAA and the other considerable problem is cost effectiveness if used routinely, especially in times when many health systems cope with expenses cuts. Fast way of LAA excision is use of stapler devices. This again is more suitable in case of minimally invasive procedures when it can be performed thoracoscopically [31].

Kanderian et al. reported that high percentage of patients with suture exclusion still have persistent flow into appendage and high percentage of those with stapler occlusion had a residual LAA stump > 1 cm. In their study even with excision of LAA residual stump was > 1 cm in 27% of patients [21]. Other groups reported complete exclusion in only 45% of patients using sutures [18]. It is possible that due to anatomical closeness of circumflexes artery some surgeons tend to leave more appendage tissue when amputating, having more material for safe closure. Also, simple ligation or purse string suture can be time effective sparing precious cross clamp time. It is important to note that incompletely excluded LAA can be more thromboembolic as blood is more stagnant than left alone at first place [32].

We must comment on the limitations of the study. It is a single centre observational study without any control group. The lack of a control group for comparison is a limitation of our study. We understand that a randomized controlled trial or a propensity-matched cohort study could provide stronger evidence on the effectiveness of the surgical method used for LAA amputation. However, our study aimed to assess the safety and invasiveness of the procedure and to control its effectiveness in terms of completeness of amputation. Despite the absence of a control group, our study provides valuable insights into the feasibility and safety of the cut and sew technique for LAA amputation. Further studies are needed to confirm our findings and to compare the effectiveness of different surgical methods for LAA amputation. Further on, effectiveness and completeness of LAA amputation was measured by intraoperative TEE. All studies and measurements are reproducible and measurements and analysis were performed by trained echocardiography anaesthesiologist and board-certified cardiothoracic attending surgeon blinded to patients and in accordance with the standardized echocardiography protocol. Nevertheless, TEE as such remains subjective diagnostic method dependable of examiner.

LAA amputation does not increase the risk of bleeding and do not affect the heart function. It can be performed safely and completely leaving minimal to no LAA residual stump having as such best cost effectiveness ratio of all occlusion techniques. LAA amputation should therefore be aggressively considered in applicable cases as part of routine open heart surgery.

## Data Availability

The datasets generated and analyzed during the current study are not publicly available, but are available from the corresponding author on reasonable request.

## Abbreviations

AF: atrial fibrillation
ANP: Atrial natriuretic peptide
BNP: brain natriuretic peptide
CVE: cerebro-vascular event
LAA: left atrial appendage
NYHA: New York Heart Association Class
POAF: postoperative atrial fibrillation
sPAP: systolic pulmonary artery pressure
TAPSE: tricuspid annular plane systolic excursion
TEE: transoesophageal echocardiography
TIA: transient ischemic attack
